# Leaf Functional Traits of Invasive Grasses Conferring High-Cadmium Adaptation Over Natives

**DOI:** 10.3389/fpls.2022.869072

**Published:** 2022-06-01

**Authors:** Muhammad Ilyas, Sakhawat Shah, Ya-Wen Lai, Jan Sher, Tao Bai, Fawad Zaman, Farkhanda Bibi, Monika Koul, Shabir Hussain Wani, Ali Majrashi, Hesham F. Alharby, Khalid Rehman Hakeem, Yong-Jian Wang, Shabir A. Rather

**Affiliations:** ^1^CAS Key Laboratory of Tropical Forest Ecology, Xishuangbanna Tropical Botanical Garden, Chinese Academy of Sciences, Menglun, China; ^2^College of Horticulture and Forestry Sciences/Hubei Engineering Technology Research Centre for Forestry Information, Huazhong Agricultural University, Wuhan, China; ^3^University of Chinese Academy of Sciences, Beijing, China; ^4^Hubei Insect Resources Utilization and Sustainable Pest Management Key Laboratory, College of Plant Science and Technology, Huazhong Agricultural University, Wuhan, China; ^5^Center for Integrative Conservation, Xishuangbanna Tropical Botanical Garden, Chinese Academy of Sciences, Menglun, China; ^6^Hubei Ecology Polytechnic College, Wuhan, China; ^7^CAS Key Laboratory of Tropical Plant Resources and Sustainable Use, Xishuangbanna Tropical Botanical Garden, Chinese Academy of Sciences, Menglun, China; ^8^Department of Botany, Hansraj College, University of Delhi, New Delhi, India; ^9^Mountain Research Centre for Field Crops, Khudwani, Sher-e-Kashmir University of Agricultural Sciences and Technology of Kashmir, Jammu and Kashmir, India; ^10^Department of Biology, College of Science, Taif University, Taif, Saudi Arabia; ^11^Department of Biological Sciences, Faculty of Science, King Abdulaziz University, Jeddah, Saudi Arabia; ^12^Princess Dr. Najla Bint Saud Al- Saud Center for Excellence Research in Biotechnology, King Abdulaziz University, Jeddah, Saudi Arabia; ^13^Department of Public Health, Daffodil International University, Dhaka, Bangladesh

**Keywords:** bioconcentration factor, cadmium, leaf functional traits, phytoremediation, physiological response

## Abstract

Heavy metal (HM) contamination resulting from industrialization and urbanization during the Anthropocene along with plant invasion can severely threaten the growth and adaptation of local flora. Invasive alien plant species generally exhibit a growth pattern consistent with their functional traits in non-contaminated environments in the introduced range. However, it remains unclear whether invasive alien plants have an advantage over native plants in contaminated environments and whether this growth pattern is dependent on the adaptation of their leaf functional traits. Here, we selected two congeneric pairs of invasive alien and native grasses that naturally co-exist in China and are commonly found growing in contaminated soil. To evaluate the effect of cadmium (Cd) on the structural and physiological leaf traits, we grew all four species in soil contaminated without or with 80 mg/kg Cd. Invasive plants contained significantly higher concentrations of Cd in all three organs (leaf, stem, and root). They displayed a higher transfer factor and bioconcentration factor (BCF) of shoot and root than natives, indicating that invasive species are potential Cd hyperaccumulators. Invasive plants accumulated polyphenol oxidase (PPO) to higher levels than natives and showed similar patterns of leaf structural and physiological traits in response to changes in Cd bioconcentration. The quantifiable leaf structural traits of invasive plants were significantly greater (except for stomatal density and number of dead leaves) than native plants. Leaf physiological traits, chlorophyll content, and flavonoid content were also significantly higher in invasive plants than in natives under Cd stress conditions after 4 weeks, although nitrogen balance index (NBI) showed no significant difference between the two species. Chlorophyll fluorescence parameters decreased, except for the quantum yield of photosystem II (ΦPSII) and the proportion of open photosystem II (qP), which increased under Cd stress conditions in both species. However, invasive plants exhibited higher fluorescence parameters than natives under Cd stress, and the decrement observed in invasive plants under Cd stress was greater than that in natives. High Cd adaptation of invasive grasses over natives suggests that invasive plants possess optimal leaf structural and physiological traits, which enable them to adapt to stressful conditions and capture resources more quickly than natives. This study further emphasizes the potential invasion of alien plants in contaminated soil environments within the introduced range. To a certain extent, some non-invasive alien plants might adapt to metalliferous environments and serve as hyperaccumulator candidates in phytoremediation projects in contaminated environments.

## Graphical Abstract

Higher structural and physiological leaf traits in invasive than in native plants. 
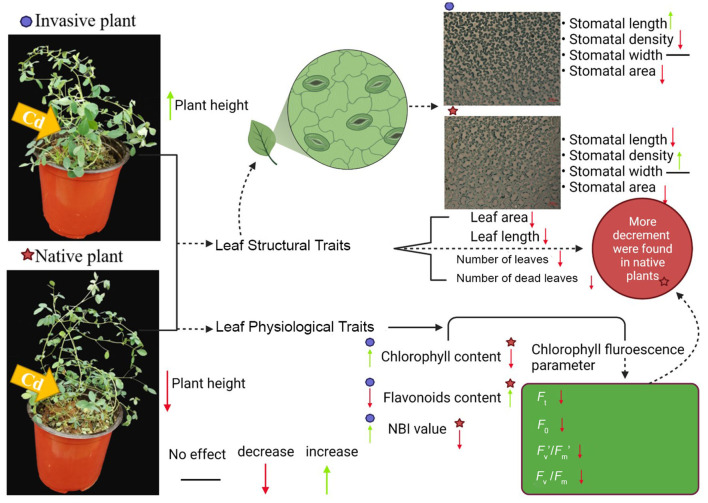

## Introduction

Heavy metal (HM) contamination, which occurred in Anthropocene, has the potential to severely threaten biodiversity and human health, especially in developing countries (Li et al., 2014; Duan et al., 2016). In the last few decades, the HM contamination of soil has spread extensively throughout the globe (Huamain et al., 1999; Dong et al., 2001). The increment of soil HM contamination is one of the common and severe threats to urban flora and fauna communities (Bi et al., 2013; Li et al., 2014). HM can greatly impact plant growth, reproduction, and migration (Deng et al., 2007). Plants lack the ability to deliberately migrate away from a polluted area, and the only way to increase their chance of survival in adverse environments is to trigger defense mechanisms and evolve tolerance-inducing genes (Chmielowska-Bak et al., 2014). When exposed to cadmium (Cd) stress, plants display signs of injury in the form of chlorosis, growth inhibition, root tip browning, and eventually death (Mohanpuria et al., 2007; Ali H. et al., 2013; Ali et al., 2014). HM stress inhibits biological processes such as photosynthesis, cell division, and water absorption, thus reducing plant growth and canopy cover (Jadia and Fulekar, 2009; Taiz and Zeiger, 2010).

Elevated Cd concentration can cause many disturbances in the morphological, anatomical, and physiological processes of plants. A recent study reported that HM reduces leaf chlorophyll index, leaf area, and various other metabolic processes in plants (Hatamian et al., 2020). Several mechanisms are involved in delaying plant development under HM stress. Cell division and leaf morphological features are adversely affected by lead (Pb) and Cd toxicity (Hatamian et al., 2020). Plants regulate their morphological, physiological, and ecological adaptation to environmental fluctuations and change the leaf functional traits under unfavorable conditions (Dwyer et al., 2014; Meng et al., 2014). Functional traits directly or indirectly affect plant adaptability through their effects on plant development, survival, and reproduction (Marteinsdóttir and Eriksson, 2014).

Plant functional traits are expected to represent general adaptation under stress (Dwyer et al., 2014; Marteinsdóttir and Eriksson, 2014; Ilyas et al., 2021). Specific leaf area (SLA) is an outstanding indicator of plant functional traits and explains the changes in plant species in response to any changes in the available resources (Kardel et al., 2010; Scheepens et al., 2010). Besides leaf size, and shape, chlorophyll content is an important functional trait of leaves, which accurately describes the plant resource use strategy (Wang et al., 2016). The competitive ability of plants for resource acquisition, especially sunlight, is influenced by plant height (Gross et al., 2007; Thomson et al., 2011). Similarly, the leaf dry weight, leaf number, and plant height significantly decreased under Cd (Farooq et al., 2016). Related or even identical environmental stresses, such as habitat filtering, can also affect the leaf functional traits (Gross et al., 2007). Furthermore, invasive plant species exhibit a higher leaf shape index than native species to increase their resource capturing efficiency and enhance their competitiveness (Liu et al., 2017). Moreover, under stress conditions, invasive plants grow significantly taller than native plants, which enables them to capture more light energy and transport more water (Ishii and Asano, 2010; Wang et al., 2017). Wang et al. (2018b) reported that HM treatments negatively affected the functional traits of invasive and native plants; however, invasive plants showed an increase in height and leaf shape index compared with native plants. Cd has a major impact on the survival and physiological efficiency of native plants. Moreover, it has been proposed that biotic stresses, such as enemy release, superior competitor, and allelopathy, benefit invasive species over resident species (Bakker and Wilson, 2001; Maron and Vilà, 2001; Keane and Crawley, 2002; Callaway and Ridenour, 2004). Many invasive plants exhibit a greater ability to use water and nutrients under stress conditions (Blicker et al., 2003), implying that the adaptation or tolerance to stressful environments may be an important trait of invasive plant species. However, well-established invasive plant populations may pay a high fitness cost during subsequent bouts of admixture with native populations (Pantoja et al., 2018). Invasive plant species demonstrate local adaptation as frequently as and at least as effectively as native plant species (Oduor et al., 2016). Previous studies confirmed that, given their functional traits, invasive plants can grow and adapt to metalliferous environments more effectively than native plants (Arredondo et al., 2006; Murtaza et al., 2017). The growth pattern and leaf functional traits of invasive alien plants are generally consistent with those observed under limited light, limited water, and limited nutrient environments or under altitude stress in the introduced range (Liu et al., 2017, 2022; Wang et al., 2017, 2019). However, whether invasive alien plants have an advantage over native plants in contaminated environments is poorly understood.

Invasive plant species invade the ecosystem and change their functions and processes by affecting nutrient cycling and reducing the biodiversity of native flora (Ehrenfeld, 2010; Simberloff et al., 2013). However, rather than getting eliminated from the ecosystem, these species are used for ecosystem restoration and the bioremediation of contaminated soil (Ewel and Putz, 2004; Pandey, 2012). In previous studies, invasive alien plant species such as *Chromolaena odorata* and *Praxelis clematidea* significantly enhanced soil nutrient levels (Koné et al., 2012; Wei et al., 2017). Therefore, some alien plant species may be useful for the restoration of degraded ecosystems. Comparatively, invasive plant species have higher adaptability to stressful environments (Ehrenfeld, 2010; Bai et al., 2020). According to previous studies, the high adaptability rate of invasive plants makes them a potentially better choice for removing soil pollutants (Tanhan et al., 2007; Sun et al., 2009; Pandey, 2012). Chlorophyll fluorescence parameters are the significant determinants of the performance and adaptability of plants in stressed environments. Fluorescence parameters such as maximum potential quantum efficiency of photosystem II (PSII; F_v_/F_m_) and quantum yield of PSII (ΦPSII) decreased under HM stress (Chen et al., 2015), and parameters including variable fluorescence (F_v_) and F_v_/F_m_ decreased in plants exposed to high Cd concentration (Li et al., 2015). In contrast, parameters including minimum fluorescence (F_0_) and non-photochemical quenching (NPQ) increased, whereas maximum fluorescence (F_m_) was not significantly affected in Purslane plants under Cd stress (Yaghoubian et al., 2016). Joshi and Mohanty (2004) reported that different concentrations of Cd had a major impact on chlorophyll a, chlorophyll b, and carotenoids contents, F_0_ and chlorophyll fluorescence in *Amaranthus caudatus* L.; however, changes in chlorophyll fluorescence parameters *F*_0_ and *F*_m_ were dependent on the experimental conditions such as the plant growth stage and metal ion exposure of plants (Joshi and Mohanty, 2004).

In this study, we aimed to understand the effect of Cd toxicity on the leaf functional traits (structural and physiological traits) of invasive and native plant species and the accumulation of Cd in different parts of these species. We hypothesized that (1) Cd stress has more negative impacts on native grasses than on invasive grasses, and invasive species exhibit greater stress resistance than native species; and (2) leaf functional traits confer better physiological response, plant adaptation, and Cd accumulation ability (i.e., high Cd adaptation) compared to invasive with grasses than to natives under stress conditions. This study provides a solid theoretical base and practical applications for the control and prevention of invasion under HM stress.

## Materials and Methods

### Species Selection and Cultivation

Two invasive (*Pennisetum purpureum* and *Paspalum dilatatum*) and two native (*Pennisetum alopecuroides* and *Paspalum distichum*) grasses, which are commonly found co-occurring in the grasslands of China, were included in this study (Supplementary Table 1). More than 100 ramets of each species were collected from the Hubei Province of China in 2014. To increase the likelihood of sampling ramets from different genets (i.e., genotypes), ramets spaced at least 500 m apart were collected. After collection, the ramets were vegetatively cultivated for 1 year in a glasshouse at Huazhong Agricultural University in Wuhan (Hubei Province, China) to produce enough new clonal fragments.

### Experimental Design

**Figure 1:**
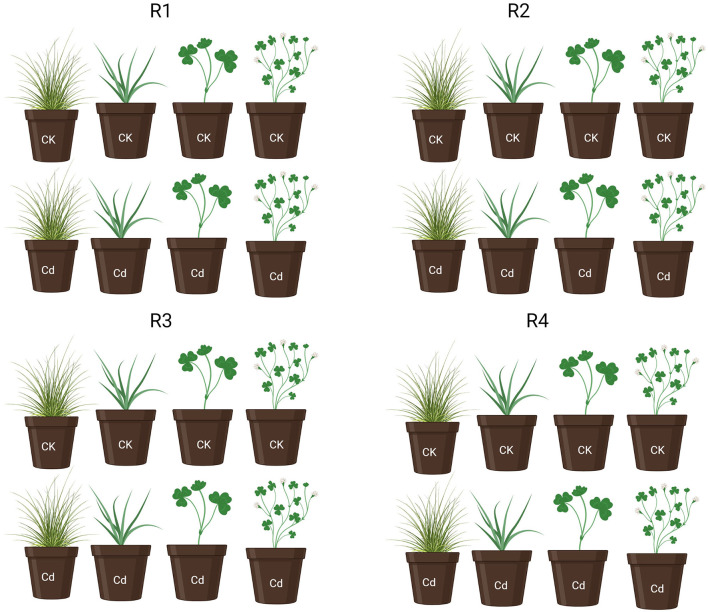
Experimental design. Notes, CK, control, Cd, cadmium stress. Experimental layout. R1-4 mean replicates.

Experiments were conducted in a plastic greenhouse at Huazhong Agricultural University, Wuhan, China. A factorial design was applied, with two levels of HM contamination in the soil (0 and 80 mg/kg). Cd was used in this study because it is one of the most important HMs found in China and was added to the soil at a concentration of 80 mg/kg (Alaboudi et al., 2018). 3.8 g of CdCl_2_·2.5H_2_O was prepared into 10 and 240 mg/ml water respectively. Before transplanting, soil samples were collected to determine the initial index of the soil. Subsequently, plants were transplanted in disposable plastic pots (30 cm diameter ×20 cm height) filled with 9.5 L of a mixture of sand and yellow-brown soil (1:1, v/v) collected from Shizishan Mountain in Wuhan, Hubei Province, China. The nutrient concentration of the soil mixture was quite low, with total N, P, and K contents of 0.23 ± 0.03, 0.32 ± 0.04, and 14.27 ± 1.25 g kg^−1^, respectively (mean ± SE, *n* = 10). Each pot contained one invasive and one native plant, which were planted in the center of the pot (Figure 1). Two weeks after transplanting, weed management and daily watering were carried out until the end of the experiment. Relevant data, including chlorophyll and flavonoid contents, nitrogen balance index (NBI), number of total leaves and dead leaves, and plant height, were measured one time a week for 8–10 weeks. Four replicate pots were used for each treatment. The experiment was terminated 10 weeks after the start of the treatments.

### Measurements of Leaf Structural Traits

To measure leaf structural traits, samples were collected at the end of the experimental period. All samples were collected at 9:00 a.m. and 11:00 a.m. on sunny days from August to September 2020. Mature leaves were randomly collected from each plant in three replicates, with each replicate containing three leaves. After collection, all leaf samples were preserved in an ice bag for transfer from the greenhouse to the laboratory for further processing. The leaves were fixed in formaldehyde-alcohol-acetic acid (FAA) for 1–2 min to measure stomatal traits. Then, the leaves were removed from the FAA and excess FAA was blotted with a filter paper. Subsequently, nail polish was applied to the adaxial and abaxial side of the leaf. After air-drying for 40 seconds, the nail polish marks were removed with transparent adhesive tape, and leaves were placed on a microscopic glass slides. To determine the density and size of the stomata in the lower epidermis, these were photographed under a light microscope (AxioPlan, Zeiss, Jena, Germany). The images were then used to determine the pore number and size. The x (long) and y (short) axes of each pore were measured using (https://imagej.net). The size of at least 30 pores was measured from each leaf sample. To determine pore density, all the pores within a 309.81-mm^2^ area were counted. Plant height and leaf length were also measured with the scale. The number of total leaves and dead leaves per plant was counted carefully. To determine the leaf area, fully expanded leaves were selected from the plants and measured using Image-Pro Plus version 6.0 (Media Cybernetics, USA). All measurements were taken in triplicate, within a 1% measurement error.

### Estimation of Chlorophyll Content, Flavonoid Content, and NBI

All representative plants in the control and Cd treatments with four replicates were selected and subjected to proximal sensing using the Dualex 4 sensor (Force-A, Orsay, France). The plant samples and sensor readings were obtained 3 weeks after planting. The Dualex values were measured on the adaxial side of leaves at six growth points: topmost, second, and third leaves before tasseling and leaves above and below the panicle as well as the panicle leaf at the tasseling stage. The Dualex readings (chlorophyll and flavonoid contents and NBI) were recorded one time a week for up to 7 weeks from leaves at the same position for each plant species.

### Measurement of Chlorophyll Fluorescence Parameters

Chlorophyll fluorescence was measured with a portable fluorometer (PAM-2500, Walz, Germany), as described previously (Genty et al., 1989). To measure fluorescence parameters, the light intensity was maintained at 200 μmol m^−2^ s^−1^. The leaf samples were dark-adapted for 30 min using leaf-clip holders (2030-B, Walz). Values of F_0_ and F_m_ were measured in dark-adapted leaves. The variable fluorescence ΦPSII quantum yield of PSII, ΦPSII qP proportion of open PSII, and ΦPSII F_v_/F_m_ maximum quantum yield of PSII were calculated as shown in Equations (1)–(3), respectively (Schreiber et al., 1986). Values of F_0_′, F_m_′, and steady-state fluorescence (F_t_) were measured using leaves adapted to actinic light (Klughammer and Schreiber, 2008).


(1) $$\begin{array}{r} \Phi \text{PSII}=\left({\text{F}}_{\text{m}}^{\prime }-{\text{F}}_{\text{t}}^{\prime }\right)/{\text{F}}_{\text{m}} \end{array}$$

(2) $$\begin{array}{r} \Phi \text{PSII}=\left({\text{F}}_{\text{m}}^{\prime }-{\text{F}}_{\text{t}}\right)/\left({\text{F}}_{\text{m}}^{\prime }-{\text{F}}_{0}^{\prime }\right) \end{array}$$

(3) $$\begin{array}{r} \Phi \text{PSII}=\left({\text{F}}_{\text{m}}-{\text{F}}_{0}\right)/{\text{F}}_{\text{m}} \end{array}$$

### Measurements of Cd Concentration and Polyphenol Oxidase Activity

The activity of PPO was measured in leaves and roots of plants treated with and without Cd stress, as described previously (Webb et al., 2014). Additionally, the Cd removal efficiency of all four plant species was quantified using a combination of shoot bioconcentration factor (shoot BCF), root BCF, and transfer factor (Wei et al., 2018; Bai et al., 2020). Shoot and root BCFs were calculated by dividing the shoot and root Cd concentrations, respectively, with soil Cd concentration at the end of the incubation period, as shown in Equations (4) and (5):


(4) $$\begin{array}{r} \text{Shoot BCF}=\left({\text{Cd}}_{\text{stem}}+{\text{Cd}}_{\text{leaf}}\right)/{\text{Cd}}_{\text{soil}} \end{array}$$

(5) $$\begin{array}{r} \text{Root BCF}={\text{Cd}}_{\text{root}}/{\text{Cd}}_{\text{soil}} \end{array}$$

where Cd_root_, Cd_stem_, Cd_leaf_, and Cd_soil_ represent Cd concentrations (mg kg^−1^) in the root, stem, leaf, and soil, respectively.

The transfer factor, which indicates the capacity of a plant to transport Cd from the root system to aboveground organs (stem and leaf), was calculated using Equation (6):


(6) $$\begin{array}{r} \text{Transfer factor}=\frac{\text{C}{\text{d}}_{\text{stem}}+\text{C}{\text{d}}_{\text{leaf}}}{\text{C}{\text{d}}_{\text{root}}} \end{array}$$

### Statistical Analysis

Two-way analysis of variance (ANOVA) was used to test the effects of soil Cd concentration (0 and 80 mg/kg) and species origin (invasive alien and native) on leaf functional (structural and physiological) traits of plants. If a significant effect of Cd contamination or species origin was detected, then Tukey's honestly significant difference (HSD) test was conducted to compare the means of different treatment combinations. Additionally, two-way repeated measures ANOVA was used to examine the effects of soil Cd concentration, species origin, and time (different experimental periods) on leaf functional traits. Data are presented as mean ± standard error (SE). Significant differences among different traits were determined using SPSS 13.0 (SPSS Inc., Chicago, IL, USA, 2016) and indicated using asterisks (^*^p < 0.05, ^**^p < 0.01, ^***^p < 0.001). All figures were drawn with OriginPro 7.0 and GraphPad Prism 8 (Graph-Pad Software Inc., San Diego, CA, USA). The relationship among different functional traits of invasive and native species was analyzed with a correlation matrix using the R software (version 4.0.3; R Development Core Team 2012).

## Results

### Effects of Cd Stress on the Morphological Traits of Invasive and Native Plants

**Figure 2:**
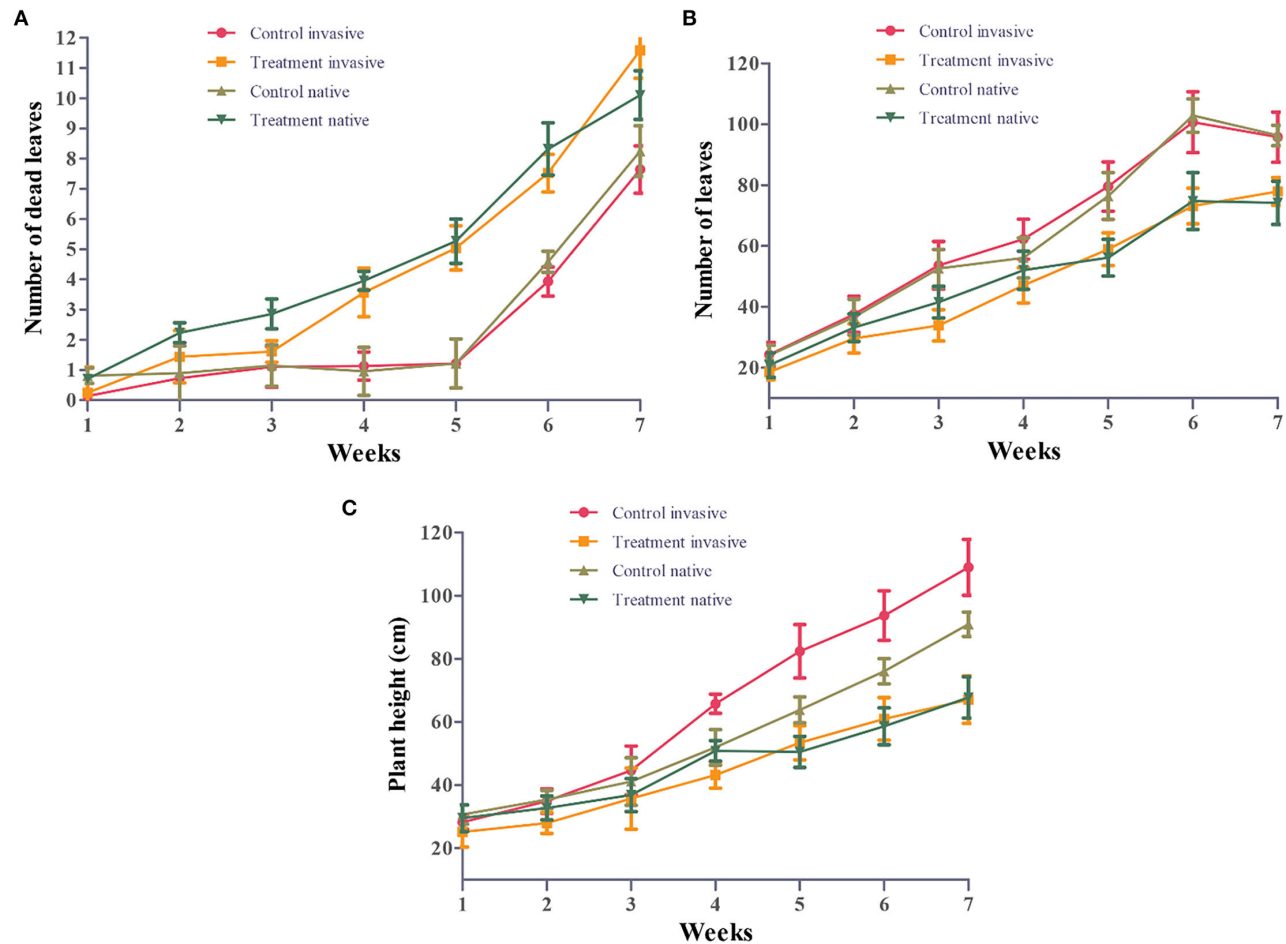
Number of leaves **(A)**, number of dead leaves **(B)** and plant height **(C)** of invasive and native plants during cadmium (Cd) stress. Control: uncontaminated soil + native and invasive: Treatment Cd contaminated soil (80 mg/kg) +native and invasive. Error bars depict the SE of the mean of four independent replicates.

Morphological traits of invasive and native plant species were significantly affected by Cd stress. However, from 3 to 5 weeks, the total leaf number of invasive and native plant species under Cd stress was significantly lower than that of control plants (*p* < 0.05). Moreover, native species showed fewer leaves than invasive species under Cd stress (Figure 2B). Cd-treated native species produced the greatest amount of leaf litter, followed by Cd-treated invasive species. Leaf litter under Cd stress was significantly higher than that in the control treatment for both invasive and native species (*p* < 0.05). However, in the Cd stress treatment, the leaf litter of native species increased from 3 to 7 weeks, whereas that of invasive plant species increased from 5 to 7 weeks (Figure 2A). During the experimental period, the plant height of invasive and native species was similar between the control and Cd stress treatments from 1 to 3 weeks but dramatically decreased from 4 to 7 weeks. The height of invasive and native plant species under Cd stress was significantly lower than that of their control counterparts (*p* < 0.05). Under Cd stress, the plant height of invasive species was greater than that of native species, indicating that the height of native plants was affected by soil contamination to a greater extent than that of invasive plants (Figure 2C). These findings suggest that leaf litter and leaf abscission are higher in native plants than in invasive plants in a metalliferous environment. This indicates that invasive species are more tolerant to HM stress and can potentially survive in a metalliferous environment.

### Effects of Cd Stress on the Leaf Structural Traits of Invasive and Native Plants

#### Effects on Leaf Morphological Traits

All quantifiable leaf structural traits of invasive plants, except stomatal density and number of dead leaves, were significantly greater than those of native plants. Among the invasive and native plants, species had significant effects on all leaf and stomatal traits. Overall, the species effects were highly significant for stomatal and leaf traits (*p* ≤ 0.001). Cd stress had significant effects on leaf length, stomatal area, stomatal length, and stomatal width (*p* ≤ 0.001) but non-significant effects on leaf area and stomatal density. The interaction between species origin and Cd stress had a significant effect (*p* ≤ 0.001) on stomatal and leaf traits, except for stomatal density and leaf area (Table 1).

**Table 1:** Two-way ANOVA analysis of effects of Cd stress on leaf functional traits of invasive and native plants.

**Effect**		**Leaf area**		**Leaf length**		**Stomatal area**	
	**Df**	**F ratio**	***P***	**F ratio**	***P***	**F ratio**	***P***
Species	3	17.17	0.001***	38.60	0.001***	3.43	0.001***
Cd	1	0.20	0.658ns	10.84	0.001***	8.33	0.001***
Species*Cd	3	0.72	0.715ns	4.15	0.003*	1.43	0.001***
**Effect**		**Stomatal length**		**Stomatal width**		**Stomatal density**	
	**Df**	**F ratio**	***P***	**F ratio**	***P***	**F ratio**	***P***
Species	3	2.63	0.001***	1.33	0.001***	4.23	0.001***
Cd	1	6.73	0.001***	2.82	0.001***	0.56	0.456ns
Species*Cd	3	4.81	0.001***	7.33	0.001***	0.40	0.94ns

Cd, cadmium Significant effects are indicated in boldface as follows: ***p ≤ 0.001, *p ≤ 0.05. ns: no significance.

**Figure 3:**
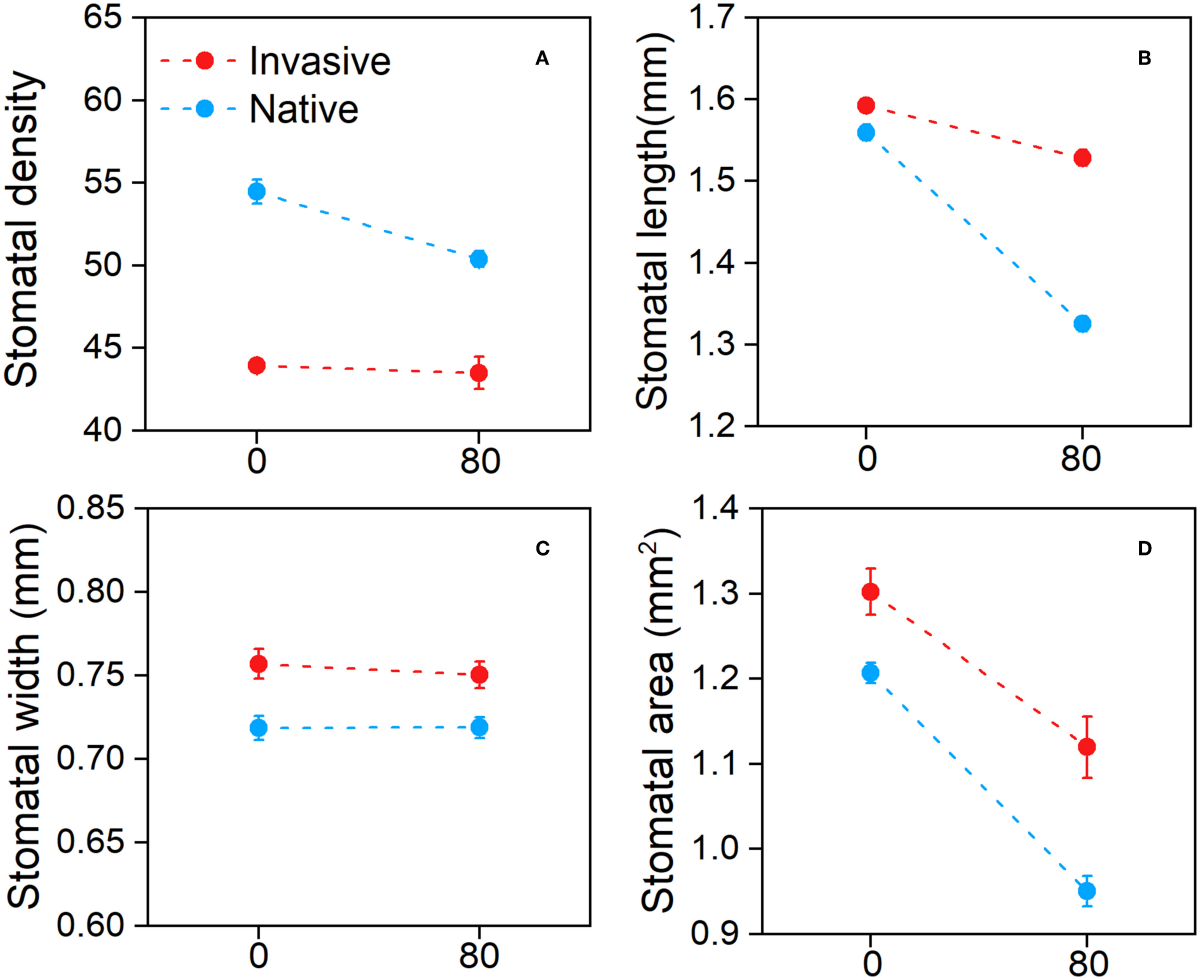
Stomatal density **(A)**, stomatal length **(B)**, stomatal width **(C)**, and stomatal area **(D)** of the invasive and native plants in response to cadmium (Cd). Values are means ± standard error (SE).

The stomatal traits of invasive and native plants showed different responses to Cd stress. The stomatal density of native species was higher in the control treatment than in the Cd treatment, whereas invasive species were similar under both conditions (Figure 3A). Moreover, the stomatal length of all species decreased following Cd treatment, and the decline observed in stomatal length was greater in native plants than in invasive plants (Figure 3B). The stomatal area data showed similar trends (Figure 3D). The stomatal width of both invasive and native plants was slightly lower in the Cd treatment than in the control treatment (Figure 3C). These results suggest that stomatal traits were affected by Cd stress, and the stomatal traits of invasive plant species respond better than that of native species in metalliferous environments.

**Figure 4:**
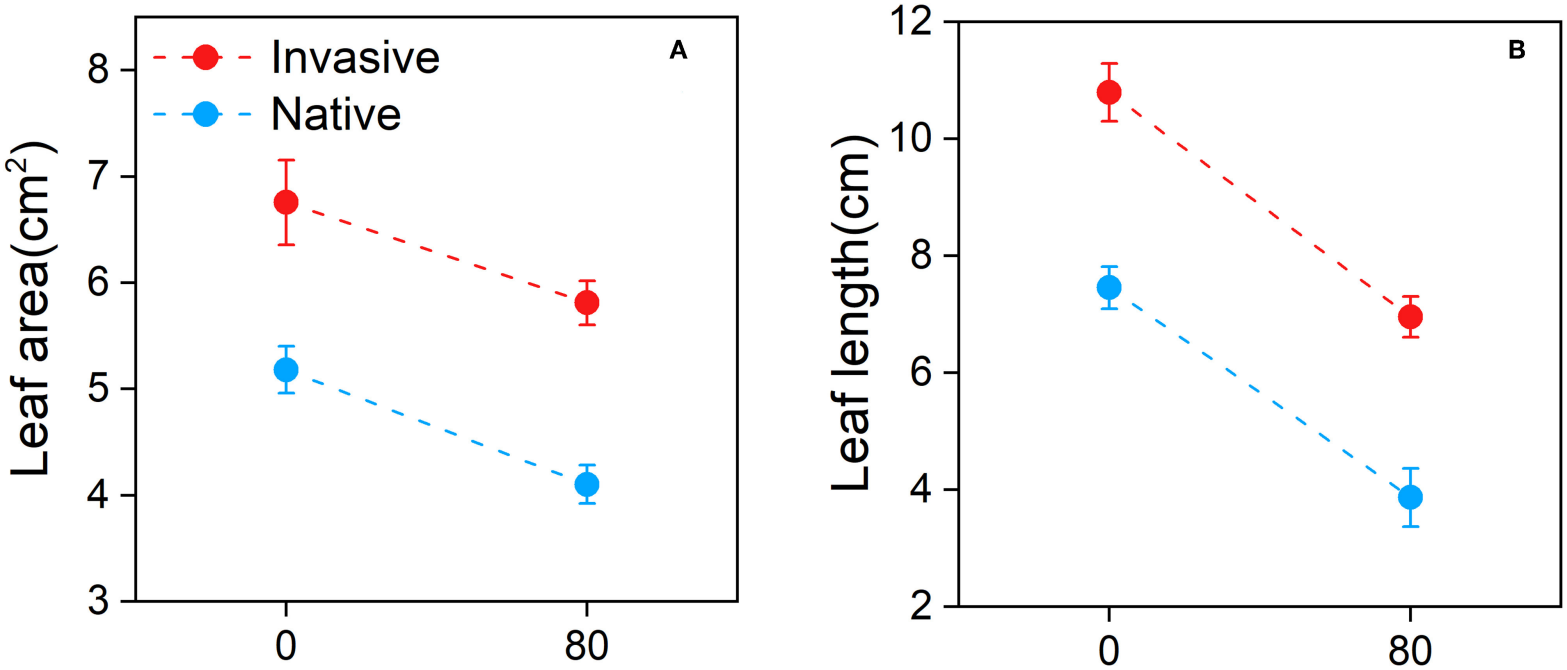
Leaf area **(A)** and leaf length **(B)** of the invasive and native plants in response to cadmium (Cd). Values are means ± standard error (SE).

Overall, the leaf area and leaf length of invasive and native species were lower under Cd stress than under control conditions (Figure 4). Under Cd stress, the leaf area of native plants was lower than that of invasive plants (Figure 4A). The leaf length data showed similar trends (Figure 4B). These results suggest that the leaf traits of native plants are affected by Cd stress to a greater extent than those of invasive plants.

#### Effects on Stomatal Morphology

**Figure 5:**
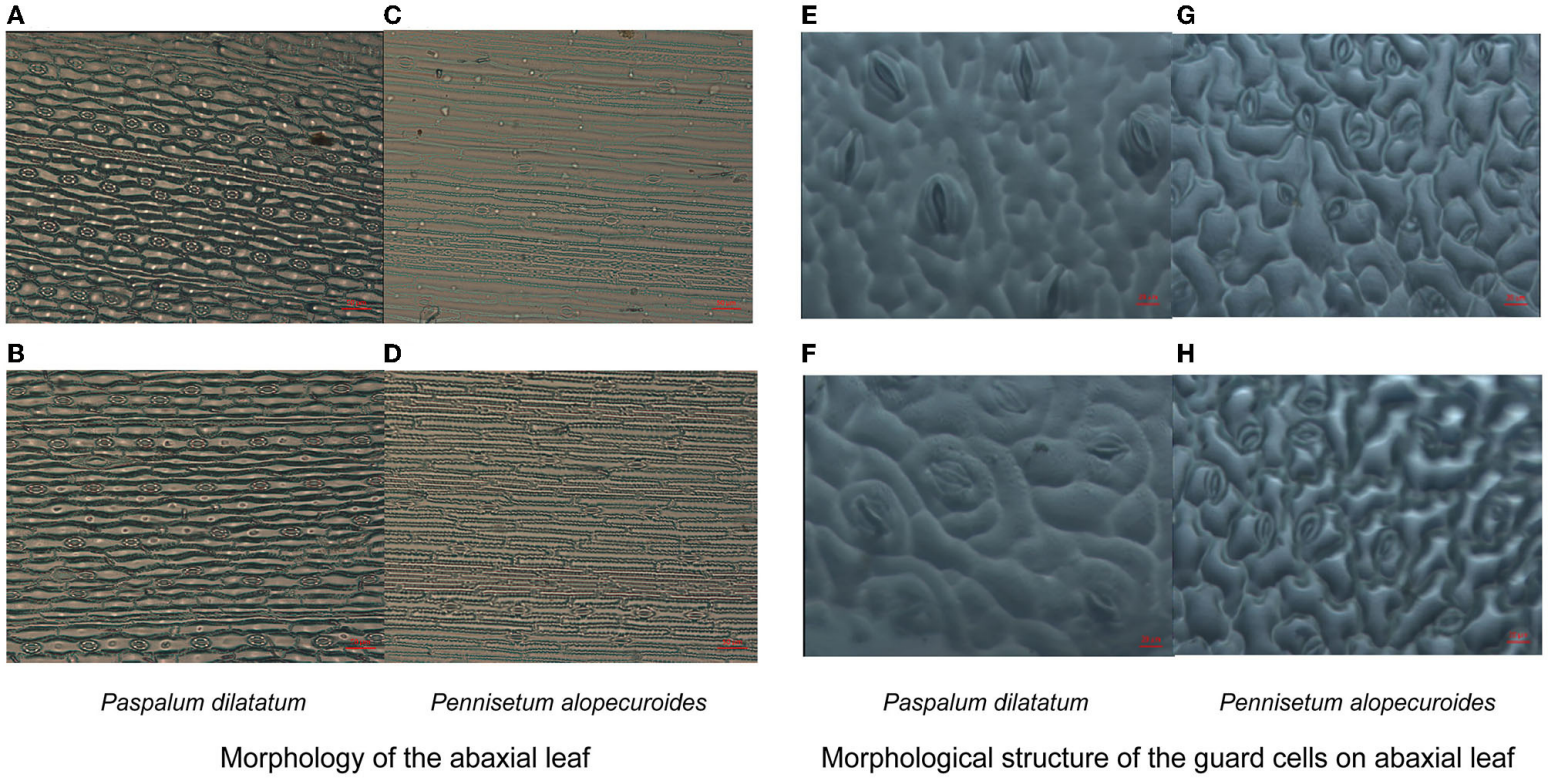
Morphology of the abaxial leaf **(A–D)** and structure of the guard cells on abaxial surface **(E–H)** of the invasive (*Paspalum dilatatum*) and native plant (*Pennisetum alopecuroides*) in control and Cd stress treatments. Images were taken by light microscope for stomatal density with 50-μm scale and for guard cells with 20-μm scale.

Exposure to Cd stress-induced clear morphological changes in the shape of epidermal cells on the abaxial leaf surface in invasive and native plants (Figure 5); however, no visible changes were observed in stomatal depth (i.e., how deep the stomata are inserted in the leaf epidermis). Under Cd stress, the stomata guard cells were affected more in native plants than in invasive plants. Additionally, Cd stress decreased stomatal length and width in invasive and native plants, although the decrease in both these parameters was greater in native plants than in invasive plants (Figure 5). These results demonstrate that the morphology of epidermal and guard cells changes under Cd stress, which increases stomatal density but decreases stomatal length, width, and area.

### Effects of Cd Stress on the Physiological Parameters of Invasive and Native Plants

**Figure 6:**
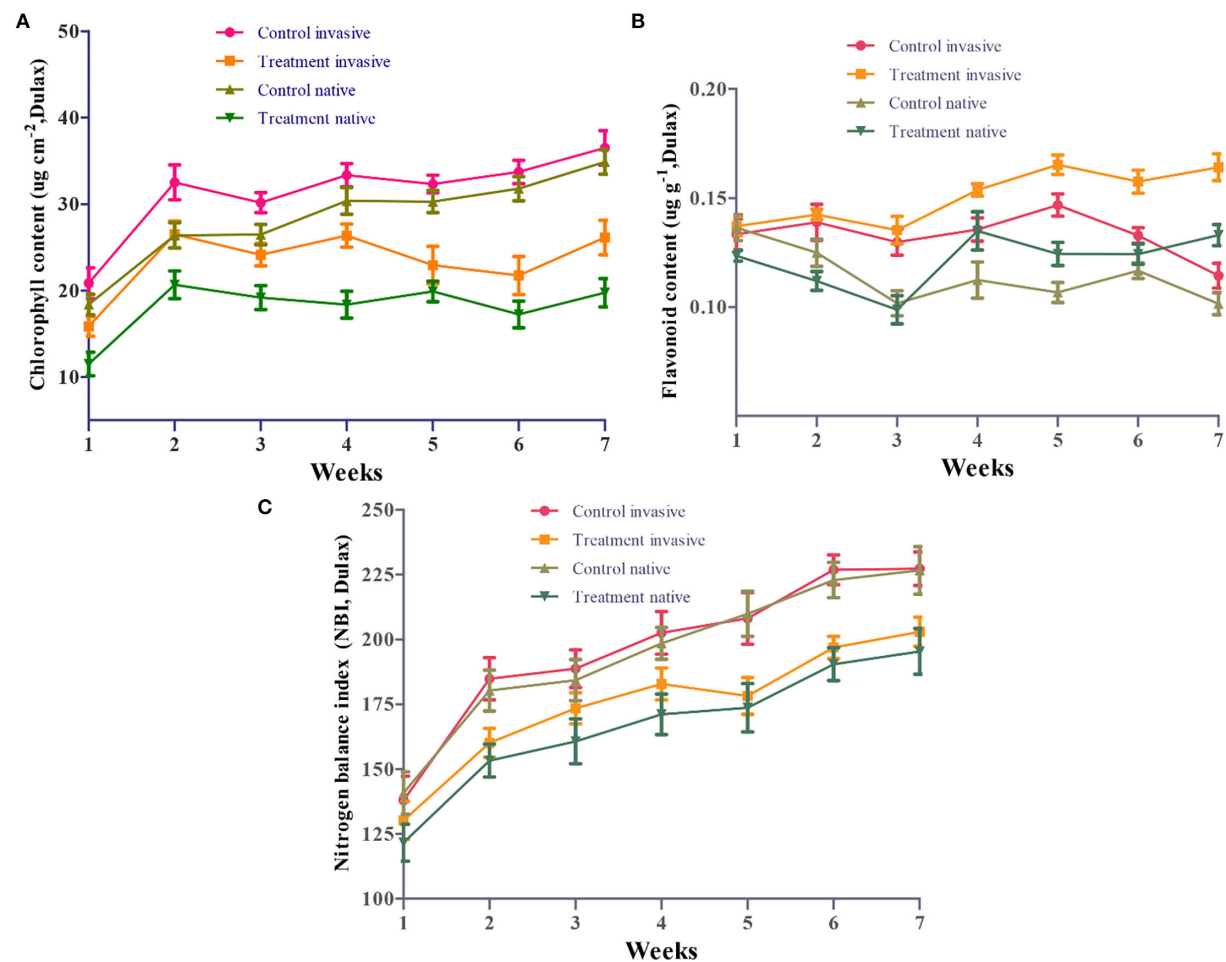
Effect of cadmium (Cd) on chlorophyll content **(A)**, flavonoids content **(B)** and NBI **(C)** of leaves in invasive and native plants during stress conditions. Control: uncontaminated soil + native and invasive plants: Treatment Cd contaminated soil (80 mg/kg) +native and invasive. Error bars depict the SE of the mean of four independent replicates.

Under Cd stress, the leaf chlorophyll and flavonoid contents of invasive species were significantly higher than those of native species after 4 weeks, although the NBI showed a significant difference between the two groups of plant species. We also investigated the effects of Cd stress on the leaf chlorophyll content of invasive and native species at different time points. The results showed that chlorophyll content of both plant groups was lower under Cd stress than under control conditions at different time points (1–7 weeks; Figure 6A). At 2–3 weeks, chlorophyll content showed a greater reduction in native species than in invasive species. The chlorophyll content of invasive and native plant species was significantly lower under Cd stress than under control conditions. Under Cd stress, the flavonoid contents of invasive and native plants increased dramatically from 1 to 7 weeks. The flavonoid content of invasive species was higher under Cd stress than in the control treatment and was higher than that of native plants under both Cd stress and control conditions (Figure 6B). The NBI values of invasive and native plants fluctuated during the experimental period. The NBI values of Cd-treated plants were lower than those of control samples. During week 1, the NBI values showed no significant difference among treatments. From weeks 2–7, the NBI values of Cd-treated native and invasive plants were lower than those of their control counterparts. However, the NBI values of invasive species were significantly higher than those of native species at some time points, particularly during weeks 3 and 4 (Figure 6C). These findings suggest that Cd stress decreased the chlorophyll content and NBI values of both invasive and native plant groups but had a greater negative impact on the flavonoid content of native species than that of invasive species.

### Effects of Cd Stress on the Fluorescence Parameters of Invasive and Native Plants

The chlorophyll fluorescence parameters of native and invasive plants decreased under Cd stress; the only exceptions were ΦPSII and qP, which increased under Cd stress. The values of fluorescence parameters were higher in invasive species than natives under Cd stress.

#### Effects on Chlorophyll Fluorescence Parameters

**Figure 7:**
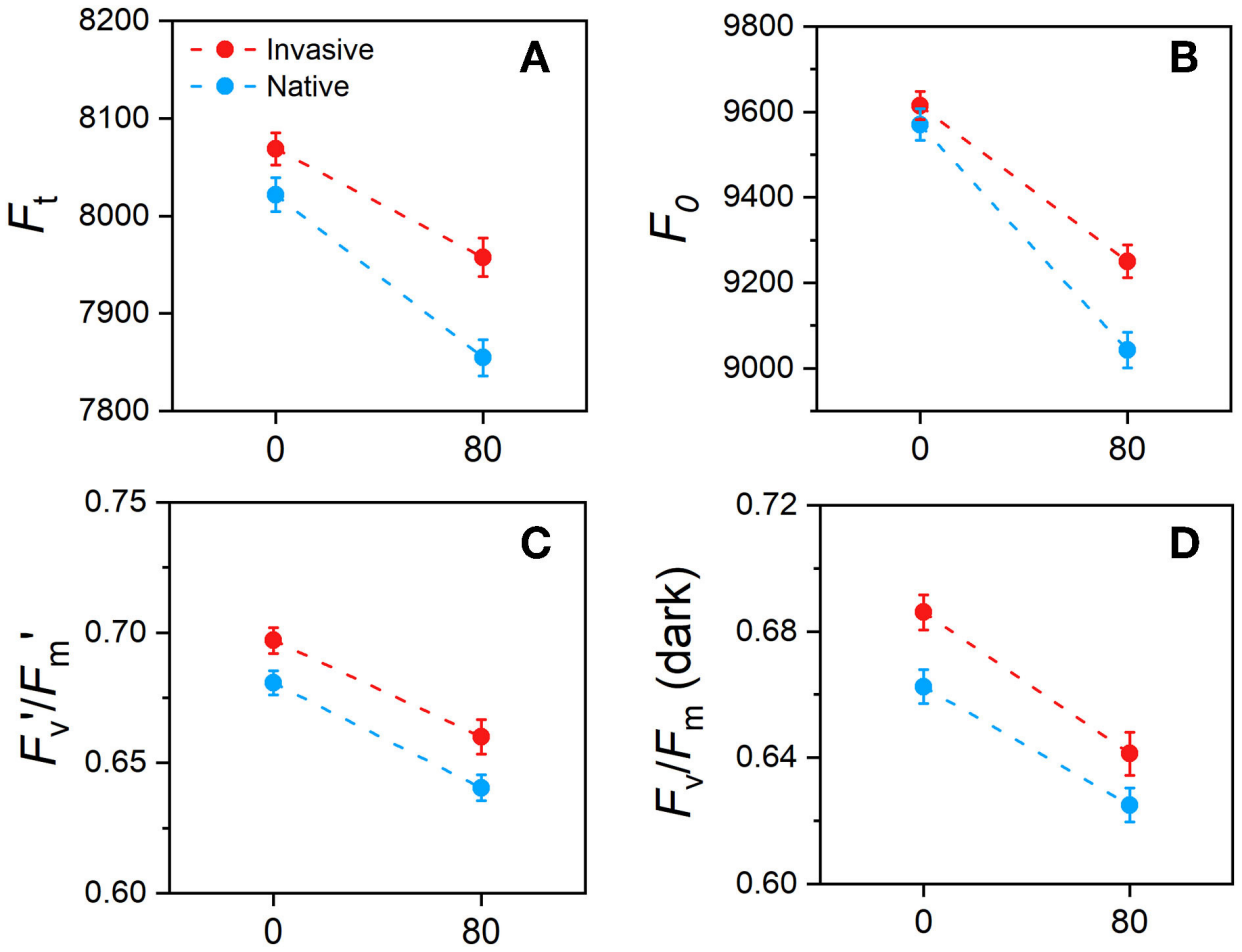
Changes in chlorophyll fluorescence parameters in young-mature (i.e., just after reaching their final size) leaves of invasive and native plants during long term stress and acclimation in response to cadmium (Cd) treatment **(A)** F_t_, ground fluorescence in the light-adapted state **(B)** F_0_, ground fluorescence in the dark-adapted **(C)** F_v_′/F_m_′, maximum chlorophyll fluorescence in the light-adapted **(D)** F_v_/F_m_ maximum chlorophyll fluorescence in the dark-adapted state. Values are means ± standard error (SE). Notes, 0, control 80, Cd concentration mg/kg.

Chlorophyll fluorescence parameters of invasive and native plants were significantly affected by Cd stress (Figures 7A,B). Values of F_t_ in invasive and native plants were lower under Cd stress than under control conditions. The F_0_ values of invasive and native plants were similar in the control treatment and decreased in the Cd treatment; however, the magnitude of reduction was greater in invasive plants than in native plants. Under Cd stress conditions, values of F_v_′/F_m_′ and F_v_/F_m_ decreased in both plants, whereas the higher decrement was found in native plants than in invasive plants (Figures 7C,D). This finding shows that invasive plants exhibit greater photosynthetic efficiency than native plants under Cd stress.

#### Effect on Photochemical and Non-Photochemical Chlorophyll Fluorescence Parameters

**Figure 8:**
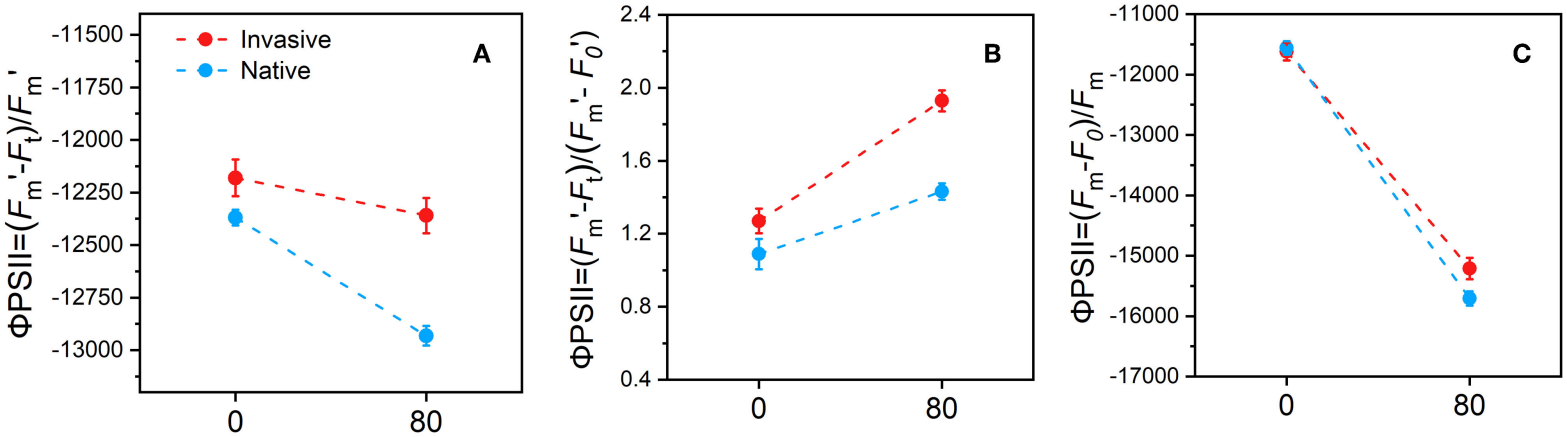
Changes in chlorophyll fluorescence parameters in young-mature (i.e., just after reaching their final size) leaves of invasive and native plants during stress and long-term acclimation in response to cadmium (Cd) treatments **(A)** ΦPSII quantum yield of photosystem II **(B)** ΦPSII qP proportion of open photosystem II **(C)** ΦPSII F_v_/F_m_ maximum quantum yield of photosystem II. Values are means ± standard error (SE).

Values of ΦPSII and F_v_/F_m_ of invasive and native plants were lower under Cd stress than under control conditions. Both parameters showed a greater decline in native plants than in invasive plants under Cd stress (Figures 8A–C). In comparison, an improvement in Cd treatment in both plant species was identified in the proportion of open photosystem II, and a further increase was detected in invasive plants simultaneously. These findings suggest that the chlorophyll fluorescence parameters of native plants are more negatively affected by Cd stress than those of invasive plants.

#### Correlation Between the Chlorophyll Fluorescence Parameters of Native and Invasive Plants

**Figure 9:**
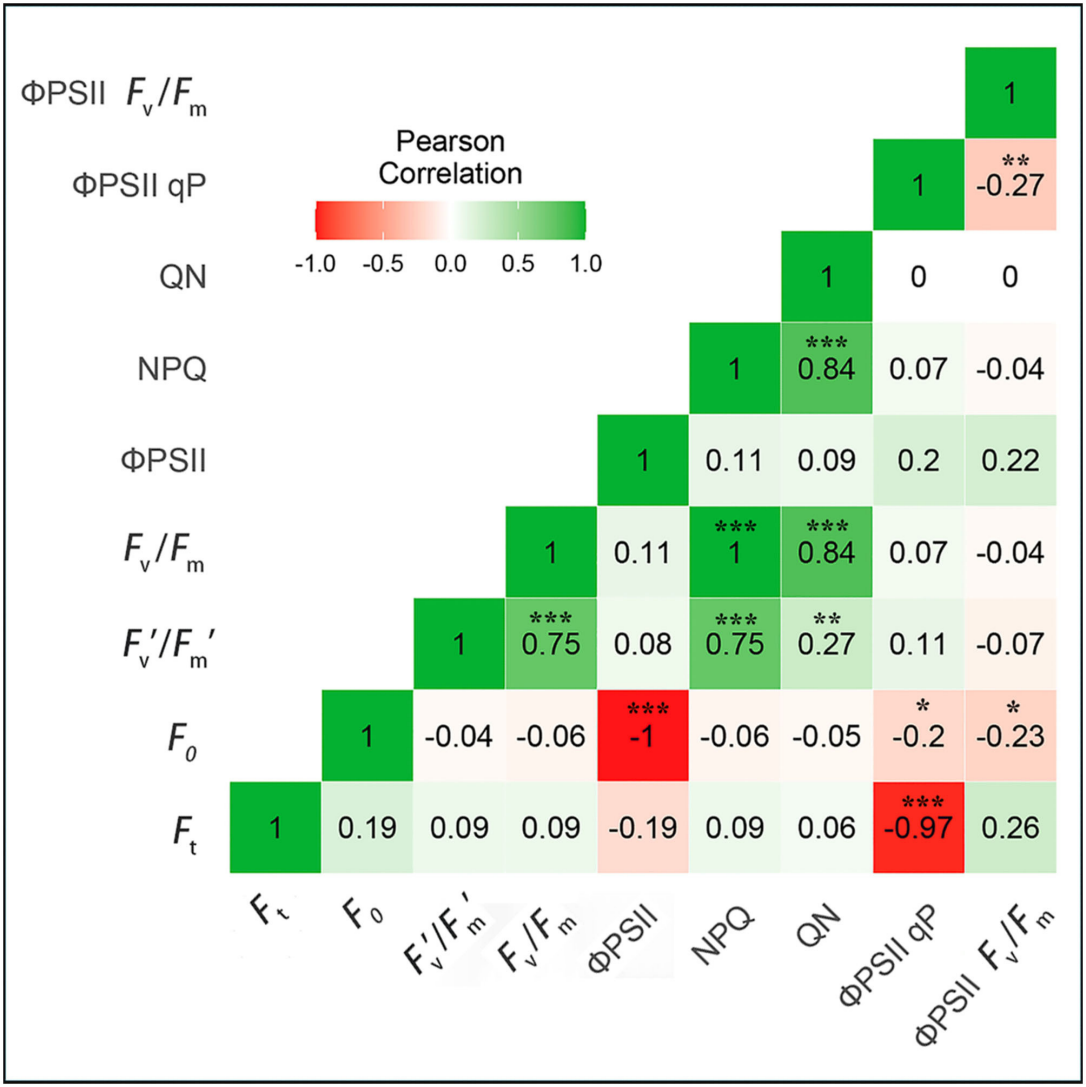
Correlation matrix of chlorophyll fluorescence parameters in young-mature leaves of invasive and native plant species, the labels were similar in Table 1. The colored gradient legends represent coefficients of correlation r values from +1.0 (green) to −1.0 (red). The significant correlation at a level (p^***^ <0.001), (p^**^ <0.001), (P^*^ <0.05). All coefficients were computed by the Pearson correlation for possible pairs of variables in the matrix.

The photochemical and non-photochemical chlorophyll fluorescence parameters, including F_v_′/F_m_′-NPQ, F_v_/F_m_-QN, and NPQ-QN, showed significant positive correlations (1, 0.84, and 0.84, respectively) between invasive and native plants. Additionally, two chlorophyll fluorescence parameters, F_0_- ΦPSII and F_t_- ΦPSII qP, showed extremely negative correlations (−1 and −0.84, respectively) between invasive and native plants. Thus, the results of correlation analysis revealed that some chlorophyll fluorescence parameters were positively correlated, whereas others were negatively correlated between invasive and native plants under Cd stress conditions (Figure 9).

### Response of Leaf Functional Traits of Invasive and Native Plants to Cd Stress

The exposure of invasive and native plants to Cd stress in greenhouse experiments resulted in a significant change in stomatal and leaf traits (Table 2). The leaf area of invasive species, *Pennisetum purpureum schum* (13.977 ± 0.970 cm^2^) and *Paspalum dilatatum* (6.229 ± 0.459 cm^2^), was greater than that of native species, *Pennisetum alopecuroides* (5.745 ± 0.577 cm^2^) and *Paspalum distichum* (3.879 ± 0.372 cm^2^). The leaf length of *Pennisetum purpureum* was the highest (28.150 ± 1.488 cm), while that of *Paspalum distichum* was the lowest (6.133 ± 1.484 cm). Similarly, *P. purpureum* showed the highest stomatal area (1.59 ± 0.120 mm^2^), and *P. distichum* showed the lowest leaf area (0.8713 ± 0.122 mm^2^). By contrast, stomatal density was the highest in *P. distichum* (48.167 ± 5.954 mm^2^) and the lowest *in P. dilatatum* (24.500 ± 5.549 mm^2^). The highest stomatal length and width (1.7110 ± 0.077 and 0.9180 ± 0.0301 mm, respectively) were observed in *P. purpureum*. In contrast, the lowest values of stomatal length (1.393 ± 0.077) and width (0.670 ± 0.040 mm) were observed in *P. alopecuroides* and *P. distichum*, respectively. Overall, all leaf functional traits of the two invasive plant species were higher than those of the two native plant species, except stomatal density. This suggests that invasive plants showed better adaptation to metalliferous conditions than native plants.

**Table 2:** The effects of Cd stress on leaf functional traits of each invasive and native plant.

**Plant species**	**Leaf area**		**Leaf length**		**Stomatal area**	
	**CK**	**Cd stress**	**CK**	**Cd stress**	**CK**	**Cd stress**
*Pennisetum purpureum* (invasive)	15.543 ± 0.992a	13.977 ± 0.970a	27.241 ± 1.318a	28.150 ± 1.488a	1.171 ± 0.086ab	1.590 ± 0.120a
*Pennisetum alopecuroides* (native)	5.245 ± 0.474c	5.745 ± 0.577c	11.757 ± 0.823bc	9.350 ± 1.483c	1.146 ± 0.108ab	1.152 ± 0.1220c
*Paspalum dilatatum* (invasive)	6.434 ± 0.451b	6.229 ± 0.459b	13.799 ± 1.132b	11.533 ± 1.438b	1.049 ± 0.096b	1.166 ± 0.120b
*Paspalum distichum* (native)	5.776 ± 0.336c	3.879 ± 0.372d	11.526 ± 1.215bc	6.133 ± 1.484d	1.343 ± 0.109a	0.871 ± 0.122d
**Plant species**	**Stomatal density**		**Stomatal length**		**Stomatal width**	
	**CK**	**Cd stress**	**CK**	**Cd stress**	**CK**	**Cd stress**
*Pennistem purpureum* (invasive)	50.333 ± 5.373b	44.000 ± 5.949b	1.461 ± 0.047b	1.7110 ± 0.077a	0.832 ± 0.027a	0.918 ± 0.030a
*Pennisetum alopecuroides* (native)	49.124 ± 4.977b	42.500 ± 5.9549b	1.392 ± 0.034b	1.393 ± 0.077c	0.822 ± 0.019a	0.828 ± 0.030ab
*Paspalum dilatatum* (invasive)	27.252 ± 1.242c	24.500 ± 5.549c	1.292 ± 0.021c	1.410 ± 0.097b	0.663 ± 0.018c	0.808 ± 0.020ab
*Paspalum distichum* (native)	62.231 ± 5.551a	48.167 ± 5.954a	1.547 ± 0.053a	1.289 ± 0.079d	0.775 ± 0.019b	0.670 ± 0.040d

*Data points are mean ± standard error; different letter was (a, b, c, d) shown as significant differences among different plants were determined by a multiple comparisons Tukey's test*.

### Cd Bioaccumulation and Translocation in Different Organs of Invasive and Native Plants

**Figure 10:**
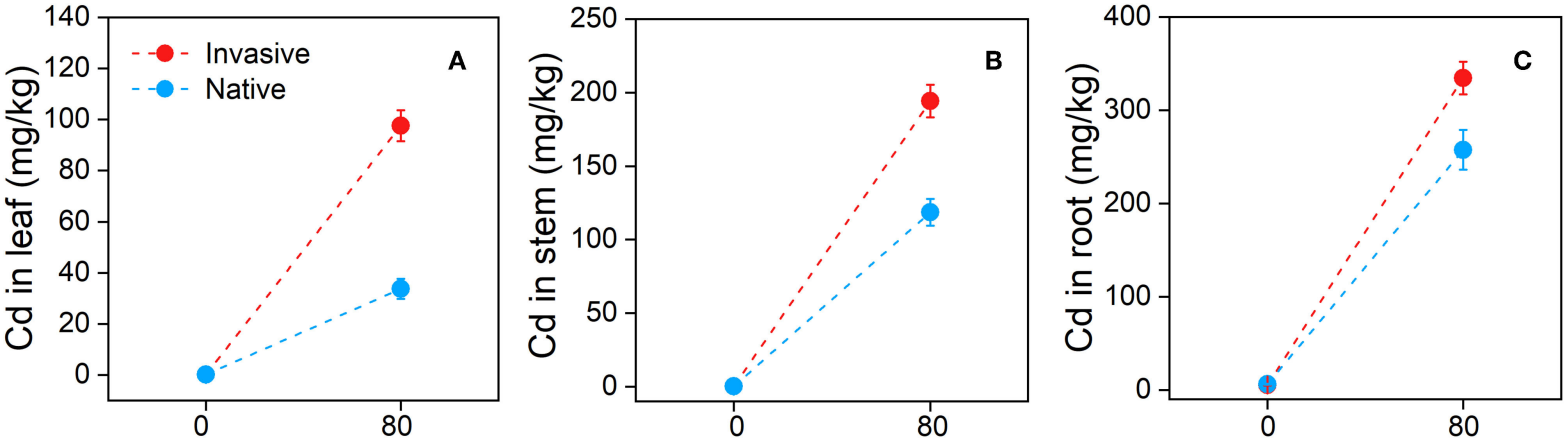
Cd (Cadmium) concentrations in leaf **(A)**, stem **(B)**, and root **(C)** of the two group of invasive and native species under Cd 80 mg/kg level. Values are means ± standard error (SE).

Invasive plant species contained a significantly higher concentration of Cd in all three organs (leaf, stem, and root) and showed higher transfer factor and shoot and root BCFs than natives, indicating that invasive species are potential Cd hyperaccumulators. Moreover, invasive plants accumulated a higher level of PPO to adapt to Cd stress than natives. At the end of the Cd treatment, the leaf Cd concentration of invasive plants was higher (97.62 ± 6.02 mg^−1^) than that of native plants (33.72 ± 3.9 mg^−1^) (Figure 10A). Similarly, the stem and root tissues of invasive species contained more Cd (194.20 ± 11.16 and 334.43 ± 17.44 mg^−1^, respectively) than those of native plants (118.67 ± 9.09 and 257.46 ± 21.35 mg^−1^, respectively) (Figures 10B,C). Surprisingly, the highest difference in Cd concentration between invasive and native plants was found in the leaf compared with other plant parts (Figure 10).

**Figure 11:**
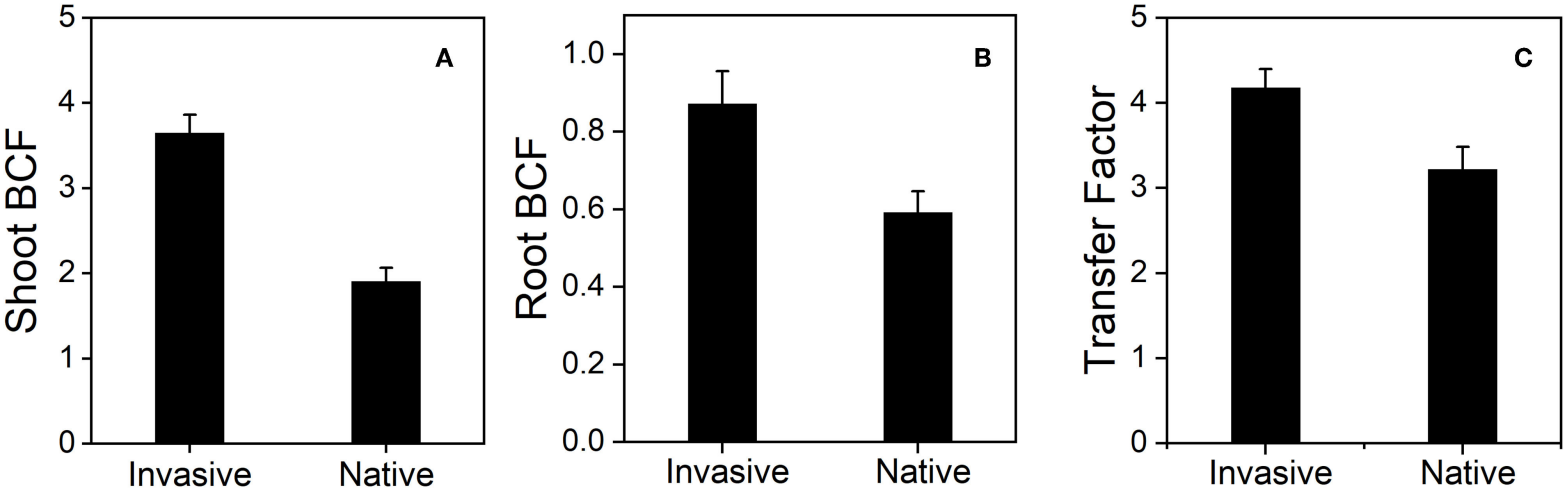
Shoot BCF (bioconcentration factor) **(A)**, Root BCF (bioconcentration factor) **(B)**, and transfer factor **(C)**, the two group of invasive and native species under Cd 80 mg/kg level. Values are means ± standard error (SE).

The shoot BCF of invasive plants (3.64 ± 0.21) was higher than that of native plants (1.90 ± 0.16) under Cd stress and decreased with the increase in soil Cd concentration in both plants (Figure 11A). Similarly, the root BCF and average transfer factor of invasive species (4.18 ± 0.21 and 0.87 ± 0.08, respectively) were greater than those of native species (3.21 ± 0.26 and 0.59 ± 0.05, respectively) (Figures 11B,C). These results suggest that invasive plant species are either Cd tolerant or Cd bioaccumulators.

**Figure 12:**
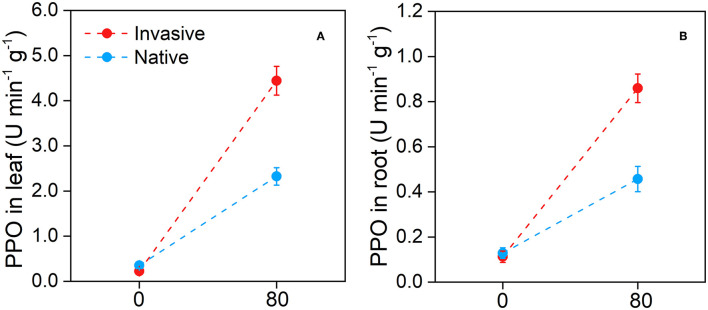
Polyphenol oxidase in leaf **(A)** and in root **(B)** in the two group of invasive and native species under Cd 80 mg/kg level. Values are means ± standard error (SE).

Compared with the control, the Cd stress treatment increased activity of PPO in the leaf and root tissues of invasive and native plants. Under Cd stress, PPO enzyme activity in the leaf of invasive plants (4.44 ± 0.31) was higher than that in the leaf of native plants (2.32 ± 0.19) (Figure 12A). Similarly, PPO enzyme was active in the invasive plant root (0.85 ± 0.06) which was higher than that in the native plant root (0.45 ± 0.05) under Cd stress conditions. Overall, PPO enzyme activity in the leaf and root tissues of invasive plants was significantly higher than that in the corresponding tissues of native plants in contaminated soil. Therefore, this study suggests that invasive plants exhibit more effective defense against stresses, such as pathogens, HM, and herbivores, than native plants.

## Discussion

Our study showed that Cd stress significantly decreased the leaf functional traits and altered the physiological parameters of both groups of plants, which showed a different response to Cd stress. Overall, invasive plant species showed better growth and higher adaptation of leaf functional (structural and physiological) traits to Cd stress than native plants.

### Response of Plant Physiological Parameters to Cd Toxicity

Chlorophyll is an important pigment required for maintaining plant growth. In this study, we investigated the effects of Cd toxicity on the chlorophyll content of invasive and native plant species at different time points for up to 7 weeks. The Cd treatment significantly decreased the chlorophyll content of both plant groups compared with the control (Figure 6A). This result is consistent with a previous study (Qian et al., 2009), which showed that Cd stress decreased plant chlorophyll and carotenoid contents because of the inhibitory effect of Cd on enzymes involved in pigment biosynthesis. Additionally, the chlorophyll content of leaves decreased significantly upon Cd application, which is consistent with previous studies showing that Cd inhibited chlorophyll biosynthesis and induced a form of senescence (Fang et al., 1998; Nada et al., 2007; Qian et al., 2009; Gill et al., 2012). Thus, the reduction in chlorophyll content of leaves indicated that Cd stress is a major factor responsible for plant injury. In this study, the negative effect of Cd on leaf chlorophyll content was less in invasive plants than in native plants, which suggests that invasive plants have high physiological adaptation to Cd stress. This result was also in line with the higher Cd accumulation in invasive plants, particularly in the roots. Similarly, *Ageratina adenophora*, a widely invasive plant species, can quickly accumulate Cd in its roots in metal-contaminated areas (Lux et al., 2011; Dai et al., 2020). These results indicate that invasive plants exhibit greater resistance to high Cd levels than native plants. Besides, Cd serves as a non-biological inducer of flavonoid synthesis in plants (Ibrahim et al., 2017). Previous research has shown that Cd present in the soil has a major impact on the content of flavonoids in the leaves of *Robinia pseudoacacia* seedlings, implying that the effect of Cd on flavonoid biosynthesis is species-specific. Furthermore, an important association between total flavonoid and soluble sugar contents in leaves revealed that Cd regulates the main metabolites and increases the total flavonoid content of leaves (Zhang et al., 2021). Similarly, flavonoid content increased dramatically at different time points during the experimental period (1–7 weeks). However, invasive plant species showed a greater increment in flavonoid content than native species (Figure 6B). The effect of Cd on flavonoid synthesis is species-dependent (Gondor et al., 2014; Zoufan et al., 2020). As a result, our findings indicate that invasive plant species with higher total flavonoid content and activity can endure Cd stress better than native plant species. Enrichment of HM in the root affects the accumulation of N, which further alters the NBI (Yadav et al., 2015). Consistent with this finding, the NBI values of invasive and native plants were reduced under Cd stress in this study (Figure 6C). The effect of change in plant photosynthetic activity on NBI values has been observed at the elevated level of HM in soil (Chapin III and Eviner, 2003). The NBI values are closely linked to C and N metabolism, and these nutrients allow plants to potentially growth and development (Zheng, 2009). Therefore, these results indicate that NBI can be used to predict plant growth.

### Response of the Leaf Functional Traits of Invasive and Native Plants to Cd Stress

The height and other morphological characteristics of plants play the important roles in determining their competitive ability in stress environments (Gross et al., 2007; Thomson et al., 2011). Our results showed that invasive plants grew taller than native plants when exposed to Cd stress (Figure 2C), which is consistent with the previous studies (Wang et al., 2018a,b). Greater height may confer a higher competitive ability to invasive plants for resource acquisition, especially for sunlight, one of the most important ecological factors required for plant growth, reproduction, and survival (Meng et al., 2014; DeMalach et al., 2017). Consequently, invasive plants exhibited superior functional traits, such as plant height and leaf shape in a metalliferous environment, which facilitated their spread and successful invasion. Furthermore, the number of leaves of invasive and native plants decreased with the increase in Cd concentration, and native plants showed a greater reduction in leaf number than invasive plants (Figure 2A). High Cd concentrations result in brownish or yellowish leaves and dry shoots, resulting in leaf litter, which is positively associated with plant biomass production (Gomes et al., 2011; Mirshekali et al., 2012). In a previous study, high Cd concentration in soil caused chlorosis, development inhibition, and leaf senescence (Mohanpuria et al., 2007). Similarly, we observed that high Cd concentration increased the senescence rates of leaves in both native and invasive species, although to a greater extent in native plants than in invasive plants (Figure 2B). Moreover, Cd stress affected the leaf traits to a greater level in native plants than in invasive plants (Figures 4A,B). As recorded in studies on *Avena sativa, Hordeum vulgare, Brassica campestris*, and *Apium graveolens*, high HM content in soil changed the morphological and functional traits of leaves, reducing plant growth and biomass (Gross et al., 2007). Additionally, under HM stress, plants exhibited phytotoxicity symptoms such as chlorosis, leaf necrosis, and decreased production of plant roots, stems, and leaves (Bini et al., 2000). Therefore, our results indicate that leaf traits of invasive plants ensure better survival in metalliferous conditions compared with native plants (Table 2). Similarly, invasive plants exhibited superior leaf functional traits and performed better than native plants under HM stress, which could accelerate their subsequent invasion ability by improving resource acquisition (Bai et al., 2020).

### Response of the Physiological Fluorescence Parameters of Invasive and Native Plants to Cd Stress

Plant growth is commonly inhibited because of reduction in leaf photosynthetic rate under stress conditions. This inhibition of photosynthesis is caused by the damage to chlorophyll fluorescence parameters (Tanyolaç et al., 2007; Rodríguez-Serrano et al., 2009). In this study, chlorophyll fluorescence parameters, including F_v_′/F_m_′ and F_v_/F_m_, of invasive and native plant species, were significantly affected by Cd stress (Figure 7). High foliar Cd concentration dramatically decreased the chlorophyll fluorescence parameters in a previous study (Baumann et al., 2009). Consistently, values of F_t_, F_0_, F_v_/F_m_, and F_v_′/F_m_′ decreased significantly under Cd stress conditions in this study (Figures 7A,D). Furthermore, previous studies showed that F_0_ reduced PSII (photochemical ability) and was correlated with the leaf chlorophyll content (Calatayud et al., 2006; Fu et al., 2012). Therefore, a greater decline observed in the chlorophyll fluorescence parameters of native plants compared with invasive plants indicates that physiological traits of invasives are tolerant to Cd stress.

The ΦPSII values represent the performance and photochemistry of plants at different photosynthetic photon flux density (PPFDs) (Maxwell and Johnson, 2000; Baker and Rosenqvist, 2004). F_v_/F_m_, qP, and NPQ have been extensively used to investigate PSII activities in plants (Liu et al., 2014). In our study, plants exhibited relatively low values of F_v_/F_m_ and ΦPSII in the Cd treatment, indicating that electron transfer at the acceptor side of PSII was impeded under Cd stress. This suggests that Cd stress causes great damage to the photosynthetic apparatus in leaves and increases ΦPSII and qP (Figures 8A,C). However, we found that chlorophyll fluorescence parameters including (F_v_′/F_m_′ – NPQ), (F_v_/F_m_ – QN), and (NPQ – QN) were significantly positively correlated under Cd stress, whereas (F_0_ – ΦPSII) and (F_t_ – ΦPSII qP) were extremely negatively correlated (Figure 9). Previous studies also reported negative correlations among qP, NPQ, ΦPSII, and NPQ (Massacci et al., 2008; Fu et al., 2012). Our results are consistent with a previous study on cucumber (Jin et al., 2017). Moreover, the reduction in NPQ and QN values, accompanied by an increase in ΦPSII and qP values, in the Cd stress treatment was in line with the results of a previous study (Gururani et al., 2015). This approach could be used to increase the photochemical competence of plants and protect them from photo-oxidation under stress conditions. Therefore, our results indicate that the photosynthetic parameters of plants are severely affected by Cd stress.

### Response of the Morphological and Structural Traits of Invasive and Native Plants to Cd Stress

Cd stress had a significant effect on stomatal traits, i.e., stomatal area, length, and width (*p* ≤ 0.001) (Table 1). Previous research has shown that plants in metalliferous environments have less stomatal areas than those in non-metalliferous environments (Khosropour et al., 2019). We found that the stomatal traits of invasive and native species were negatively affected by Cd stress, although invasive plants showed a better response than native plants (Figure 3). The greater decline in the stomatal density and stomatal area of native plants indicates that the development and growth of stomata were affected to a greater extent in native plants than in invasive plants in metalliferous environments (Dineva, 2004). Additionally, in contaminated environments, changes in stomatal traits and structure have been reported in various plant species (Ghouse et al., 1980; Baruah et al., 2014). Stomatal length was found to be negatively linked to increasing stress exposure in a recent study (Aasamaa et al., 2001). In this study, both native and invasive plant groups showed a clear increment in stomatal density under Cd stress, although the increase in stomatal density was greater in native than in invasive plants (Figure 5). This result demonstrates that contamination levels alter stomatal density as an adaptation (Verma et al., 2006; Shiv and Ila, 2014). Moreover, previous studies demonstrated that stomatal density increased whereas the length and width of stomata decreased under HM contamination (Alves et al., 2008; Gostin, 2009). Therefore, this study results suggest that the morphological structure of epidermal cells and guard cells changes under Cd stress.

### Cd Bioaccumulation and Translocation in Invasive and Native Plants

Our results suggest that invasive species could potentially be used for the phytoremediation of Cd-contaminated soil. Invasive plants showed high bioaccumulation of Cd (Figure 10) and high shoot BCF, root BCF, and transfer factor (Figure 11). The high shoot and root BCFs are the two important indicators that reflect the pertinence of species for phytoremediation. The transfer factor shows the competence of plants to transfer HM from belowground organs to aboveground plant parts (Marques et al., 2009; Rascio and Navari-Izzo, 2011). However, in previous studies, plant species with shoot BCF and transfer factor > 1 have been accepted as HM hyperaccumulators. In our study, invasive plants exhibited greater BCF and transfer factors than natives. The shoot and root BCF were 3.64 for invasive plants and 1.90 for native plants, whereas the transfer factor values were 0.87 and 0.59 for invasive and native species, respectively (Figure 11). The shoot and root BCF were greater than the commonly recognized threshold (Marques et al., 2009; Ali B. et al., 2013). Additionally, higher PPO activity in invasive plant species suggest that plants exhibit great defense in response to Cd stress (Figure 12). Similarly, in a previous study, *Salicornia* plants were found to be more resistant to HM stress than other plants based on the PPO activity (Adamski et al., 2012; Khalilzadeh et al., 2020). These results suggest that invasive plant species are more effective in bioaccumulating and translocating Cd and avoiding Cd stress.

## Conclusion

This study revealed that invasive plant species could be employed as potential Cd hyperaccumulators as they had higher Cd levels in all three plant parts (leaf, stem, and root) and showed higher shoot and root BCF than native plant species. Similarly, the increased activity of PPO in invasive plants may comprise the adaptive defense system against Cd toxicity, which shows that invasive plants are hyperaccumulators of HMs and more resistant to metalliferous conditions. The quantifiable leaf functional traits of invasive plants were significantly higher than that of native plants under Cd stress which indicates that invasive plants can spread further as their ability to capture resources increases. This is particularly true as they have more access to sunlight and soil nutrients as the length of the plant parts shows increasing trend both in root and shoot system. Furthermore, chlorophyll fluorescence parameters provide additional insights into the responses of invasive and native plants to HM and can identify a variety of conditions suitable for the partial reversal of photo-inhibitory damage. However, native plants showed a greater decline in chlorophyll fluorescence parameters than invasive plants. This implies that elevated Cd concentrations reduce the photosynthetic ability of native plants and impact their physiological and biochemical processes. We conclude that invasive plants grow better and show greater adaptation to metalliferous environments than native plants. Based on our results, we propose that *Pennisetum purpureum* and *Paspalum dilatatum* are the bioaccumulators of Cd and can be recommended for plantation in Cd-contaminated soil. Our study further emphasizes that the potential invasion by alien plants in contaminated soil environments is occurring within the introduced range. Therefore, alien non-invasive plants and native plants should be recommended to facilitate land phytoremediation in contaminated environments.

## Data Availability Statement

The original contributions presented in the study are included in the article/Supplementary Material, further inquiries can be directed to the corresponding author/s.

## Author Contributions

MI, Y-JW, and MK: conceptualization. MI, TB, and Y-JW: methodology. SS and Y-WL: software. JS, FB, FZ, and SW: validation. SAR and MI: formal analysis, investigation, and writing—original draft preparation. AM, HFA, KRH, and SAR writing, reviewing, and editing. Y-JW: resources, supervision, project administration, and funding acquisition. All authors contributed to the article and approved the submitted version.

## Funding

This research was supported by NSFC (32171510, 31770449, 32071527), the Fundamental Research Funds for the Central Universities (2662020YLPY016), and Hubei Forestry Scientific Research Project (2020-LYKJ1).

## Conflict of Interest

The authors declare that the research was conducted in the absence of any commercial or financial relationships that could be construed as a potential conflict of interest.

## Publisher's Note

All claims expressed in this article are solely those of the authors and do not necessarily represent those of their affiliated organizations, or those of the publisher, the editors and the reviewers. Any product that may be evaluated in this article, or claim that may be made by its manufacturer, is not guaranteed or endorsed by the publisher.
