# Supplementary material for: Leaf Functional Traits of Invasive Grasses Conferring High-Cadmium Adaptation Over Natives
Source: Front Plant Sci. 2022 Jun 1;13:869072. doi: 10.3389/fpls.2022.869072 (PMC9202595; doi:10.3389/fpls.2022.869072)
Supplement: Supplementary file 1 [file Table_1.DOCX]

**Table S1** Information of invasive alien and native plant species used in the experiment.

| Species | Family | Status | Native range | Introduction time |
| --- | --- | --- | --- | --- |
| *Paspalum dilatatum* Poir. | Gramineae | Invasive alien | South America | 1862 |
| *Pennisetum purpureum* Schum. | Gramineae | Invasive alien | Africa | 1960s |
|  |  |  |  |  |
| *Paspalum distichum* L. | Gramineae | Native | Asia | *-* |
| *Pennisetum alopecuroides* (L.) Spreng. | Gramineae | Native | Asia | *-* |

Status and habitat information are based on the databases of Alien Species of China (IASC) (www.iplant.cn/ias) and Flora of China (www.efloras.org).
